# Perceived Risk of Insect-Based Foods: An Assessment of the Entomophagy Attitude Questionnaire Predictive Validity

**DOI:** 10.3390/insects12050403

**Published:** 2021-04-30

**Authors:** Francesco La Barbera, Mario Amato, Roberto Fasanelli, Fabio Verneau

**Affiliations:** 1 Department of Political Science, University of Naples Federico II, Via Rodinò 22/A, 80138-Naples, Italy; francesco.labarbera@unina.it (F.L.B.); verneau@unina.it (F.V.); 2 Department of Social Science, University of Naples Federico II, Vico Monte di Pietà, 80138-Naples, Italy; roberto.fasanelli@unina.it

**Keywords:** feed, direct entomophagy, indirect entomophagy, EAQ, incremental validity

## Abstract

**Simple Summary:**

Feeding the world in a sustainable way has become a very important topic in both scientists’ and policymakers’ agenda, in order to limit the repercussions of traditional food systems on planet resources. Edible insects have already proven to be safe for the environment and with a high nutritional value, but Westerners are still reluctant towards this novel food. Therefore, the aims of this paper are twofold, and were tested on a sample of 202 Italian consumers. On one hand, we aimed to further develop a recently validated psychological instrument, the Entomophagy Attitude Questionnaire (EAQ), by combining it with a measure of perceived risk. On the other hand, we wanted to test whether different animals fed with insects would be accepted by consumers. Our results clearly show that the perceived risk does not significantly improve the predictive validity of EAQ, while, with respect to the second aim, we found that beef and pork reared with insects were less accepted than fish and poultry.

**Abstract:**

Insects are a promising alternative protein source and their possible integration in the human diet has been extensively studied, also with reference to the degree of consumer acceptability and the main factors determining reluctance among Western consumers. Several studies have also proposed the use of protein meals derived from insects in animal feed as a possible way to promote the development of the insect chain. Consumer attitudes, perceived risks, and intention to eat insect-based foods have been extensively researched, yet the relationships between those factors are still unclear. On a sample of 202 Italian consumers, the present research used the Entomophagy Attitude Questionnaire (EAQ) to analyse the degree of acceptability of insects as food and meat obtained from animals raised on insect-based feeds with a specific focus on the role of attitudes and perceived risk. The research also evaluated the differences in acceptability between different types of animals fed with insects. The results show that the intention to engage in entomophagy is significantly correlated with all three of the EAQ’s subscales, as well as with perceived risk. However, the effect of perceived risk does not significantly improve the predictive validity of EAQ with respect to the intention to eat insect-based food. The results also show that the degree of acceptability for different insect meal-reared animals changes among consumers: beef and pork are characterized by a lower degree of acceptability, while poultry and fish are more accepted by consumers.

## 1. Introduction

There is a growing consensus that, in the near future, food production will increase in order to fulfil the needs of a growing population and the changing of dietary habits [1]. Therefore, in an attempt to provide a plausible answer to the problem, during the last decade, a global academic focus has been the development of alternative protein sources and innovative and safe food processing methods, with less negative effects on the atmosphere, soil, and oceans [2,3,4].

Among the new alternative sources of protein for human nutrition, edible insects represent a viable opportunity; in most edible insects, the contents of saturated fatty acid ratio are low (less than 40% of the total fatty acids) [5], whereas their protein content and/or essential amino acid is even higher than in plant proteins such as cereals and legumes [6]. In the same fashion, according to Oonincx and de Boer [7], to produce 1 kg of edible protein, mealworms required only 10% of the land needed for beef production. Furthermore, edible insects have the advantage of being able to be farmed vertically [8]. For an updated overview of insects and food consumption sustainability, see [9].

Over time, scholars’ interest towards insects as feed has also greatly increased. Processed Animal Proteins (PAP) derived from insects have been recently authorized by the Regulation (EU) No (2017/893) of the European Commission for the aquaculture sector [10], and the EU Commission envisions authorizing PAPs in pig and poultry diets in the near future [11], in order to increase the protein self-sufficiency ratio and reducing consequently the dependence on soy imports from South American countries [12]. The increase in scientific findings on the use of insect-derived proteins in animal feed and the gradual removal of the ban on the use of PAPs, at least in monogastric animals, has also stimulated research interest in consumer acceptability of insects in animal feed. In recent years, several studies have investigated the acceptability of insects as a source of protein in aquaculture and in broiler or pig farming [13,14,15].

Although the benefits of eating insects are evident, and despite the fact that edible insects have been part of the human diet for thousands of years [16], nowadays, the consumption of insect-based foods is still a niche, due to persistent biases, especially in Western countries. Many studies have been carried out to understand the psychological and practical barriers to the development of this sector in Western markets, by applying isolated and refined insect proteins to conventional foods [17,18,19].

La Barbera and colleagues recently developed and validated an instrument for measuring the specific attitude individuals holds towards entomophagy, the Entomophagy Attitude Questionnaire (EAQ) [20]. More specifically, the EAQ measures attitudes towards either direct entomophagy (i.e., eating food containing insect-based ingredients) and indirect entomophagy (i.e., eating food derived from animals reared using insect-based feed). The EAQ has been translated and cross-validated in several languages, and administered in Western and non-Western countries [21,22]. These studies show an overall negative attitude towards direct entomophagy, whereas the attitude towards indirect entomophagy seems to be better in all geographical and social contexts investigated.

This negative attitude and the generally low willingness to eat insects seems mostly due to cultural beliefs that portray insects as filthy and unhealthy animals [23], and societies that consume insects as lousy and feral [24]. In addition, scholars have highlighted the important role that safety, real risk and perceived threat could play in orienting consumers’ choices in relation to insect-based foods.

### Safety and Perceived Risk of Insect-Based Food

Starting from Regulation (EC) 178/2002 [25], the European legislation on food safety has been progressively redesigned in all its most important aspects, and today it represents one of the best-defined elements within the policies for the protection of consumers and their health, markets and society. In summary, the key elements of food safety strategy are based on four pillars: the first one is represented by the horizontal regulations on Good Practices, HACCP system, and traceability. The second pillar is represented by the European Food Safety Authority (EFSA), the third one corresponds to the Food Safety Inspection system for official controls and enforcement, and finally the fourth pillar is the Rapid Alert System for Food and Feeds (RASFF). Moreover, Reg. 178/2002, better known as the Food Law (GFL), codifies risk analysis as the core principle of the modern food safety policy. Risk analysis is based on three main components—risk assessment, risk management and risk communication.

A further step in the definition and implementation of food safety policies at European level is the new Regulation (EU) 2015/2283 on Novel Foods [26], which has been applicable since 1 January 2018, and explicitly refers to insects as food. In view of the possible introduction of insects as food and feed, EFSA produced an opinion in 2015 in the form of a risk profile, according to which the microbiological and the environmental risk of insect farming is expected to be comparable to other animal production systems when currently allowed feed materials are used as substrate [27]. More recently, in January 2021, EFSA released the first full evaluation of a product proposed as an insect-derived food. After a risk assessment procedure, EFSA concluded that mealworms do not present objective risks and are safe for human consumption [28]. Nevertheless, the reluctance of consumers to introduce insect-based foods into their diet does not depend solely or primarily on objective risk assessment, yet on their subjective perception of risk.

From a theoretical perspective, perceived risk is the evaluation of the magnitude and likelihood of the possible negative outcomes which may result from an action [29]. Adopting a marketing-oriented point of view, perceived risk is defined as a subjectively determined expectation of loss [30]. In recent years, scholars have evaluated the role of perceived risk as an inhibitor of willingness to try insects [31,32].

As we mentioned, risk perception could be defined as the individual judgment of the likelihood and the seriousness of the likely consequences of a certain choice or behaviour [29]. Nevertheless, the definition of attitude is also based on individual beliefs about the probability and value attributed to a certain consequence or outcome (see, for example [33,34]). In the rational action approach, which is the most widely used in social psychology and consumer studies, attitude towards a behaviour (e.g., eating insects) is determined by the beliefs individuals hold about the likely consequences of performing that behaviour and the value attributed to these consequences (see [35], for an overview; see also [36], for a recent application of the model).

Therefore, despite attitudes and perceived risk being related, this link has not been sufficiently investigated in the case of direct and indirect entomophagy. The aim of the current study was to assess whether adding a measure of perceived risk to established measures of attitudes would improve the prediction of individuals’ intention to eat insects (direct entomophagy) and animals fed with insects (indirect entomophagy).

A secondary aim of the study, with respect to indirect entomophagy, was to explore whether intention to engage in indirect entomophagy and perceived threat may be differently elicited by different animals.

For example, several species of fish are either omnivorous or carnivorous. This implies that, especially in the case of freshwater fish, their diet naturally includes a variety of insect species. Additionally, most farmed birds, such as chickens, turkeys and guinea fowls, include in their diet many types of invertebrates, including insects. Lastly, among bred mammals, pigs have omnivorous habits and therefore, besides vegetables, roots and fruits, they also eat insects and other animals. All ruminants, however, along with other monogastric animals such as horses and donkeys, are strictly herbivores, and only incidentally ingest insects or other invertebrates.

Research assessing consumer acceptability has found more favourable attitudes and higher willingness to eat meat from animals raised on insect-based feeds than foods containing insects or directly raw insects [20,37]. However, different degrees of acceptability have been documented for different types of meat and the factors influencing consumers’ willingness to eat meats from insect-based feed depends on animal that will feed on insects [38]. Consumers seem to be more favourable in the case of aquaculture fish, chicken meat and eggs while appearing more reluctant in the case of pork and especially in the case of cattle [37,38,39].

## 2. Materials and Methods

Between the end of July and the first week of August 2020, the Italian version of the EAQ [20] was administered to a sample of 202 Italian participants, along with two items measuring perceived threat, and two short scales measuring intentions about direct and indirect entomophagy. The research protocol was approved by the Psychological and Social Research Lab “R. Gentile” of the University of Naples Federico II. Participants were recruited through a snowball sampling on the most popular social media and were asked to fill an online questionnaire hosted on the Google Modules platform, with the only requirement being 18 years or older. The questionnaire was anonymous, and participants did not receive any payment. Eight questionnaires were dropped because participants declared to be vegetarian or vegan. The final sample consisted of 194 valid responses (122 females; M_age_ = 42.56; SD_age_ = 13.57). All participants completed an informed consent form before filling out the questionnaire. Table 1 outlines the descriptive statistics of the sample.

Attitudes towards direct and indirect entomophagy were measured using the EAQ [20], which consists of three subscales. The disgust subscale (EAQ-D) consists of five items (Cronbach’s α = 0.92); higher values indicate a negative attitude towards direct entomophagy. The interest subscale (EAQ-I) consists of three items (Cronbach’s α = 0.93); higher values indicate a positive attitude towards direct entomophagy. The third subscale (EAQ-F) is composed by two items (Cronbach’s α = 0.79); higher values indicate a positive attitude towards indirect entomophagy.

Perceived risk was measured by two items, one for perceived probability and the other for perceived seriousness. The scores on the two items were multiplicated, resulting in a single score of perceived risk [40].

Participants’ intention to eat dishes containing insects (direct entomophagy) was measured by means of three items derived by previous research [20]; the reliability of this measure proved satisfactory (Cronbach’s α = 0.93), and the items were averaged in a single score: the higher the score, the higher the intention to engage in direct entomophagy.

Four totally new items were developed for measuring participants’ intention to eat insect-fed animals (indirect entomophagy), taking into account that feeding different animals with insects could be differently perceived by consumers. Each item made explicit reference to a specific animal. However, an exploratory factor analysis (method: maximum likelihood) extracted a single factor with eigenvalue >1, explaining the 89% of total variance. The item analysis showed that the best reliable scale was the one including all four items (Cronbach’s α = 0.96). Therefore, the items were averaged in a single score, with higher values indicating higher intention to engage in indirect entomophagy.

All answers were collected on self-anchoring scales ranging from 1 (totally disagree) to 7 (totally agree). All items used in the questionnaire, together with means and standard deviations, are reported in Table 2.

In the following sections, statistical analysis was performed using SPSS 26 (IBM); the statistical comparison of correlation coefficients was carried out by R–*cocor* package [41]; the accepted level of significance in the current research was set at *p* < 0.05.

## 3. Results

Table 3 provides means and standard deviations of the study variables as well as correlations among them. In line with previous research, participants score higher on intention to engage in indirect entomophagy than in direct entomophagy; the three components of the EAQ are significantly intercorrelated; the intention to engage in direct and indirect entomophagy is significantly correlated with all three EAQ’s subscales. Perceived risk is inversely correlated with positive attitudes (interest and feed), whereas it is positively correlated with disgust. Importantly, as expected, perceived risk has a significant correlation with both intentions. Overall, from a nomological point of view, correlations are perfectly in line with theory and expectations that could be derived from previous research.

To assess whether the measure of perceived risk would improve the predictive power of the EAQ with respect to intention to engage in direct and indirect entomophagy, two stepwise regression models were run. In the first model, intention to eat insects (direct entomophagy) was regressed on the three EAQ subscales in the first step. The model explained a substantive proportion of variance (81%), and all three subscales were significantly associated with intention. Perceived risk was entered in the second step as an explanatory variable, but the additional variance explained in participants’ intention was virtually zero; in addition, the effect of perceived risk on intention was not significant. Finally, participants’ sex and age were entered in the third step as statistical controls; they did not contribute to explain additional variance in intention, and none of them was significantly associated with intention. Complete results of this first stepwise regression model are provided in Table 4.

In a similar fashion, another stepwise regression was run for assessing whether perceived risk—added to the EAQ components—would increase the prediction of intention to engage in indirect entomophagy. In the first step, intention to eat animals fed with insects (indirect entomophagy) was regressed on the three EAQ’s subscales. The model explained a substantive proportion of variance (about 60%); two out of three subscales, disgust and feed, were significantly associated with intention, whereas interest was not. Perceived risk was added in the second step as an explanatory variable, but the additional variance explained by this variable (0.7%) did not yield a significant change in the overall explained variance. The effect of perceived risk on intention to engage in indirect entomophagy was not significant. In the third step, participants’ sex and age were entered as statistical controls and again they did not contribute significantly to the explained variance; none of them was significantly associated with intention as well. Complete results of the second stepwise regression model are provided in Table 5.

In line with the second aim of the study, we compared the mean scores of the four items used for measuring intention to eat animals fed with insects. Results of pairwise comparisons are summarized in Table 6. The mean scores of intentions to eat beef and pork are not different from each other, but significantly differ in their means relative to fish and poultry. Hence, a subgrouping of animals seems to emerge with respect to indirect entomophagy: beef and pork on the one side, fish on the other one, and poultry in an intermediate position.

To further investigate this aspect, we compared the correlation of these four items related to different animals with perceived risk (see Table 7). Pairwise comparisons between correlation coefficients showed that the correlation between perceived risk and intention to eat pork reared with insects was significantly higher than the correlation between perceived risk and intention to eat fish, *z* = 1.67, *p* = 0.047 [42]. No other significant difference between the correlation coefficients emerged (*p* > 0.20).

## 4. Discussion and Conclusions

The present study extends the literature on entomophagy and its acceptability with novel evidence on the relation between attitude, perceived risk, and intention to eat insects. Results show a nomologically sound pattern of relationships between those variables: in line with previous research, we found significant correlations between positive and negative attitudes towards entomophagy on the one side, and intention to engage in entomophagy on the other side. In line with previous research, perceived risk is also correlated with intention towards both direct and indirect entomophagy. However, when entered together into a predictive model, the association between perceived risk and intention did not determine any incremental validity. In other words, the three-factor model of the EAQ—which already showed a sound fit in previous research conducted in several different countries [20,21,22]—is not improved by adding a measure of perceived risk. This is of course a further confirmation of the predictive validity of this instrument, yet it also suggests that, at least in relation to the topic of entomophagy, good measures of attitudes may be sufficient for predicting consumers’ intention, thus orienting communication and policies. This is important because theoretical and methodological parsimony represents an advantage for researchers and stakeholders, especially in relation to issues that are extremely timely and rapidly changing over time.

In addition, our study confirms that the acceptance of indirect entomophagy is generally higher than the acceptance of direct entomophagy [13,20,22,43,44]. Therefore, the starting point for the development of the insect supply chain could coincide with the insects farming for the feed industry. In this way, the supply chain could move away from the current experimental and start-up dimension, increasing production volumes and taking advantage of economies of scale, which would guarantee greater efficiency and reduction of the cost structure in a lean supply chain perspective. The diffusion of insect-based feeds would also have immediate positive effects in terms of environmental sustainability and balance of trade in agro-food products. Indeed, while in the case of proteins for human use, European Union countries are self-sufficient, in the case of proteins for animal feed, the EU depends heavily on international markets. This is certainly true for soybeans and soybean meals, which are imported from South American countries. It should also be underlined that most of the imported soybean is genetically modified and that in many European countries there is still a high degree of aversion towards GMO products [45].

In line with previous research reviewed in the introduction, our study shows that different animals reared on a diet comprising insects may be perceived in different ways by consumers. Intention to eat beef and pork is lower than intention to eat poultry and fish; at the same time, perceived risk seems to be higher for pork than fish. Nevertheless, it is important to acknowledge that the magnitude of these differences is overall small, and in most cases not statistically significant. This is also clearly shown by the fact that the four items of indirect entomophagy, related to different animals reared on a diet comprising insects, still load on a single factor and form a scale characterized by a very high internal coherence (0.96). This suggests that, besides differences between animals involved, indirect entomophagy should be treated as a separate and well-distinguished latent construct with its own characteristics, relations, and antecedents, which are often shown to be different from those of other forms of entomophagy (such as eating raw insects or dishes with insect-derived ingredients) by a growing corpus of literature.

Although the results of the present study cannot be generalized for the entire Italian population due to the unrepresentativeness of the sample, the main findings allow a clear development of this line of research. Indeed, insects as feed has not received yet the growing media coverage of the entomophagy trend [46]. Therefore, since most of the general public is still unaware of the potential benefits of this alternative protein source for farm animals, future studies should focus on different communications strategies and messages trying to highlight which approach could achieve the best result in terms of acceptability by the final consumer. Furthermore, it might be of interest to test whether a significant difference of perception and intention to try/eat/buy exists between conventional fed livestock and livestock reared with a diet comprising insects. Finally, the three EAQ dimensions, jointly with other sociodemographic and behavioural measures, could be used for a market segmentation in order to profile consumers more eager to introduce in their diet meats from animals reared with a diet comprising insects.

## Figures and Tables

**Table 1:** Descriptive statistics of the sample (*n* = 194).

Characteristics	Frequency	Sample (%)
**Gender**		
Male	70	36.1
Female	121	62.4
N/A	3	1.5
**Education**		
Middle school	3	1.5
High school	47	24.2
Bachelor-level	29	14.9
Graduate	69	35.6
Post-Graduate	46	23.7
**Occupation**		
Student	26	13.4
Worker	151	77.8
Unemployed	7	3.6
Retired	10	5.2

**Table 2:** Mean values and standard deviations of the items used in the study questionnaire.

Description	Item	Mean	SD
Disgust (EAQ-D)	I would be disgusted to eat any dish with insects.	5.15	1.92
	Thinking about the flavour that a bug might have sickens me.	5.08	1.93
	If I ate a dish and then came to know that there were insects among the ingredients, I would be disgusted.	4.99	1.93
	I would avoid eating a dish with insects among the ingredients, even if it was cooked by a famous chef.	5.06	2.07
	I would be bothered by finding dishes cooked with insects on a restaurant menu.	4.52	2.17
Interest (EAQ-I)	I’d be curious to taste a dish with insects, if cooked well.	3.44	2.15
	In special circumstances, I might try to eat a dish of insects.	3.82	2.08
	At a dinner with friends I would try new foods prepared with insect flour.	3.22	2.00
Feeding animals (EAQ-F)	Using insects as feed is a good way of producing meat.	3.97	1.63
	I think it is fine to give insect-based feed to fish that are farmed for human consumption.	4.35	1.66
Risk	In your opinion, does eating insects pose a risk to human health? [Extremely unlikely/extremely likely]	3.42	1.72
Seriousness	How serious do you think the risks of eating insects could be for human health? [Not at all serious/extremely serious]	3.39	1.73
Intention direct	I am ready to try edible insect foods as soon as they are available on the market.	2.70	1.87
	I plan to try eating edible insect foods as soon as they are available on the market.	2.76	1.93
	I am ready to include edible insect foods in my diet on a regular basis as soon as they are available on the market.	2.13	1.43
Intention indirect	I am ready to eat beef from animals raised on insect feed as soon as it is available on the market.	3.82	1.90
	I am ready to eat fish reared on insect feed as soon as it is available on the market.	4.15	1.85
	I am ready to eat chicken reared on insect feed as soon as it is available on the market.	3.97	1.87
	I am ready to eat pork reared on insect feed as soon as it is available on the market.	3.83	1.92

Note: If not specified in the table, answers were collected on a 7-point scale anchored from 1 = totally disagree to 7 = totally agree; *n* = 194.

**Table 3:** Summary of the study variables intercorrelations, means and standard deviations (*n* = 194).

Measure	1	2	3	4	5	6
1. EAQ-D	4.96					
	(1.75)					
2. EAQ-I	−0.834 *	3.49				
		(1.94)				
3. EAQ-F	−0.309 *	0.421 *	4.16			
			(1.49)			
4. Perceived Risk	0.382 *	−0.343 *	−0.383 *	13.88		
				(12.36)		
5. Intention direct	−0.869 *	0.844 *	0.396 *	−0.370 *	2.52	
					(1.66)	
6. Intention indirect	−0.480 *	0.519 *	0.728 *	−0.409 *	0.523 *	3.95
						(1.78)

Note: The table shows Pearson’s *r* correlation coefficients. Diagonal cells report the means (standard deviation in parentheses). The range of values is 1–7 for all variables except for perceived risk (1–49); * = *p* < 0.001.

**Table 4:** Stepwise regression model of the intention to engage in direct entomophagy (*n* = 194).

Predictor	β	t	R^2^	ΔR^2^
Step 1			0.808	-
EAQ-D	−0.551 ***	−9.406		
EAQ-I	0.348 ***	5.664		
EAQ-F	0.088 *	2.476		
Step 2			0.808	0.000
EAQ-D	−0.548 ***	−9.153		
EAQ-I	0.348 ***	5.656		
EAQ-F	0.084 *	2.253		
Perceived Risk	−0.010	−0.280		
Step 3			0.812	0.003
EAQ-D	−0.551 ***	−9.230		
EAQ-I	0.339 ***	5.513		
EAQ-F	0.083 *	2.219		
Perceived Risk	−0.007	−0.200		
Gender	0.032	0.984		
Age	0.042	1.294		

* = *p* < 0.05; *** = *p* < 0.001.

**Table 5:** Stepwise regression model of the intention to engage in indirect entomophagy (*n* = 194).

Predictor	β	t	R^2^	ΔR^2^
Step 1			0.612	-
EAQ-D	−0.251 ***	−3.007		
EAQ-I	0.037	0.419		
EAQ-F	0.643 ***	12.769		
Step 2			0.618	0.007
EAQ-D	−0.222 ***	−2.622		
EAQ-I	0.040	0.465		
EAQ-F	0.612 ***	11.605		
Perceived Risk	−0.093	−1.793		
Step 3			0.624	0.003
EAQ-D	−0.218 **	−2.584		
EAQ-I	0.046	0.530		
EAQ-F	0.613 ***	11.630		
Perceived Risk	−0.092	−1.781		
Gender	0.018	0.393		
Age	−0.075	−1.634		

** = *p* < 0.01; *** = *p* < 0.001.

**Table 6:** Pairwise comparisons of intentions to eat animals fed with insects (*n* = 194).

		Difference (SD)	t	*p*
1	Bovine–Fish	−0.330 (1.075)	−4.276	0.000
2	Bovine–Poultry	−0.149 (1.015)	−2.052	0.042
3	Bovine–Pork	−0.005 (0.885)	−0.081	0.935
4	Fish–Poultry	0.180 (0.935)	2.688	0.008
5	Fish–Pork	0.325 (1.180)	3.835	0.000
6	Poultry–Pork	0.144 (1.002)	2.005	0.046

**Table 7:** Table of correlations of perceived risk and intention to eat different animals fed with insects (*n* = 194).

Measure	1	2	3	4	5
1. Perceived Risk	-				
2. Intention to eat Bovine	−0.383 *	-			
3. Intention to eat Fish	−0.347 *	0.837 *	-		
4. Intention to eat Poultry	−0.394 *	0.856 *	0.874 *	-	
5. Intention to eat Pork	−0.419 *	0.893 *	0.805 *	0.861 *	-

* = *p* < 0.001.

## Data Availability

Data are available upon reasonable request to contact author.
